# Malaria risk assessment and mapping using satellite imagery and boosted regression trees in the Peruvian Amazon

**DOI:** 10.1038/s41598-019-51564-4

**Published:** 2019-10-23

**Authors:** Elisa Solano-Villarreal, Walter Valdivia, Morgan Pearcy, Catherine Linard, José Pasapera-Gonzales, Diamantina Moreno-Gutierrez, Philippe Lejeune, Alejandro Llanos-Cuentas, Niko Speybroeck, Marie-Pierre Hayette, Angel Rosas-Aguirre

**Affiliations:** 1 Université de Liège, 4000 Liège, Belgium; 2 Research Institute of Health and Society (IRSS), Université catholique de Louvain, 1200 Brussels, Belgium; 3 Institute of Tropical Medicine Alexander von Humboldt, Universidad Peruana Cayetano Heredia, Lima, 15102 Peru; 4 Ministry of Development and Social Inclusion, Lima, 15047 Peru; 5 Namur Research Institute for Life Sciences (Narilis), Université de Namur, 5000 Namur, Belgium; 6 Institute of Life-Earth-Environment (ILEE), 5000 Namur, Belgium; 7 National Aerospace Development Commission, Lima, 15046 Peru; 8 University of Antwerp, 2000 Antwerp, Belgium; 9 Faculty of Human Medicine, Universidad Nacional de la Amazonía Peruana, Loreto, 160 Peru; 10 Fonds de la Recherche Scientifique (FNRS), 1000 Brussels, Belgium

**Keywords:** Diseases, Malaria

## Abstract

This is the first study to assess the risk of co-endemic *Plasmodium vivax* and *Plasmodium falciparum* transmission in the Peruvian Amazon using boosted regression tree (BRT) models based on social and environmental predictors derived from satellite imagery and data. Yearly cross-validated BRT models were created to discriminate high-risk (annual parasite index API > 10 cases/1000 people) and very-high-risk for malaria (API > 50 cases/1000 people) in 2766 georeferenced villages of Loreto department, between 2010–2017 as other parts in the article (graphs, tables, and texts). Predictors were cumulative annual rainfall, forest coverage, annual forest loss, annual mean land surface temperature, normalized difference vegetation index (NDVI), normalized difference water index (NDWI), shortest distance to rivers, time to populated villages, and population density. BRT models built with predictor data of a given year efficiently discriminated the malaria risk for that year in villages (area under the ROC curve (AUC) > 0.80), and most models also effectively predicted malaria risk in the following year. Cumulative rainfall, population density and time to populated villages were consistently the top three predictors for both *P. vivax* and *P. falciparum* incidence. Maps created using the BRT models characterize the spatial distribution of the malaria incidence in Loreto and should contribute to malaria-related decision making in the area.

## Introduction

In spite of the investment in control and prevention allocated by the Peruvian government over the last decades, malaria due to both *Plasmodium vivax* and *P. falciparum* remains a significant public health issue in the country. In 2015, 61,856 malaria cases were reported^1^, representing ~15% of the total reported cases in the Americas and showing a continuous increase since 2012^2,3^. The Amazonian department of Loreto is the most affected area, accounting for 95% of national cases despite being home of only 3.5% of the Peruvian population^2^.

Loreto is a hypoendemic malaria area^4,5^, but malaria transmission in the department is highly heterogeneous, with some areas in remote Amazonia having entomological inoculation rates (EIRs) near those reported in Africa. This heterogeneity creates opportunities for targeted interventions, provided high-risk hotspots are first identified^3,6^. Since the 1960s, malaria risk maps have been essential for planning interventions^7–9^. Recently, electronic geographic information systems and remote sensing have improved these maps by providing an array of environmental and social data collected through passive and active satellite sensors^10–13^.

The main factors responsible for the variation in malaria transmission in the Amazon are environmental characteristics facilitating larvae breeding sites (temperature, precipitation, natural and human-made water bodies) and mosquito resting places (surrounding vegetation, deforestation) for the primary malaria vector of *P. vivax* and *P. falciparum*, *Anopheles darlingi*^14–17^. Social factors also influence malaria transmission by increasing exposure (forest-based economic activities)^18,19^, by contributing to delayed diagnosis and treatment (poor geographical accessibility to health facilities)^20–22^, or by limiting effectiveness of malaria interventions (lower availability and utilization of control resources and preventive measures, and inappropriate treatment-seeking behaviors)^23–25^.

Satellite data^26,27^ were used to derive environmental and social potential predictors^28–30^ of malaria risk in villages of Loreto during the period 2010–2017. The association of these predictors with malaria occurrence in the villages was analyzed using boosted regression trees (BRTs), a method that accounts for nonlinearities and interactions of variables with high predictive accuracy^31–33^, and allows for disease mapping^32,34–36^. Yearly analyses were performed to determine the relevance of predictors at local scale across the years. Maps identifying villages at highest risk were created to support the malaria decision making in Loreto.

## Methods

### Study area

**Figure 1:**
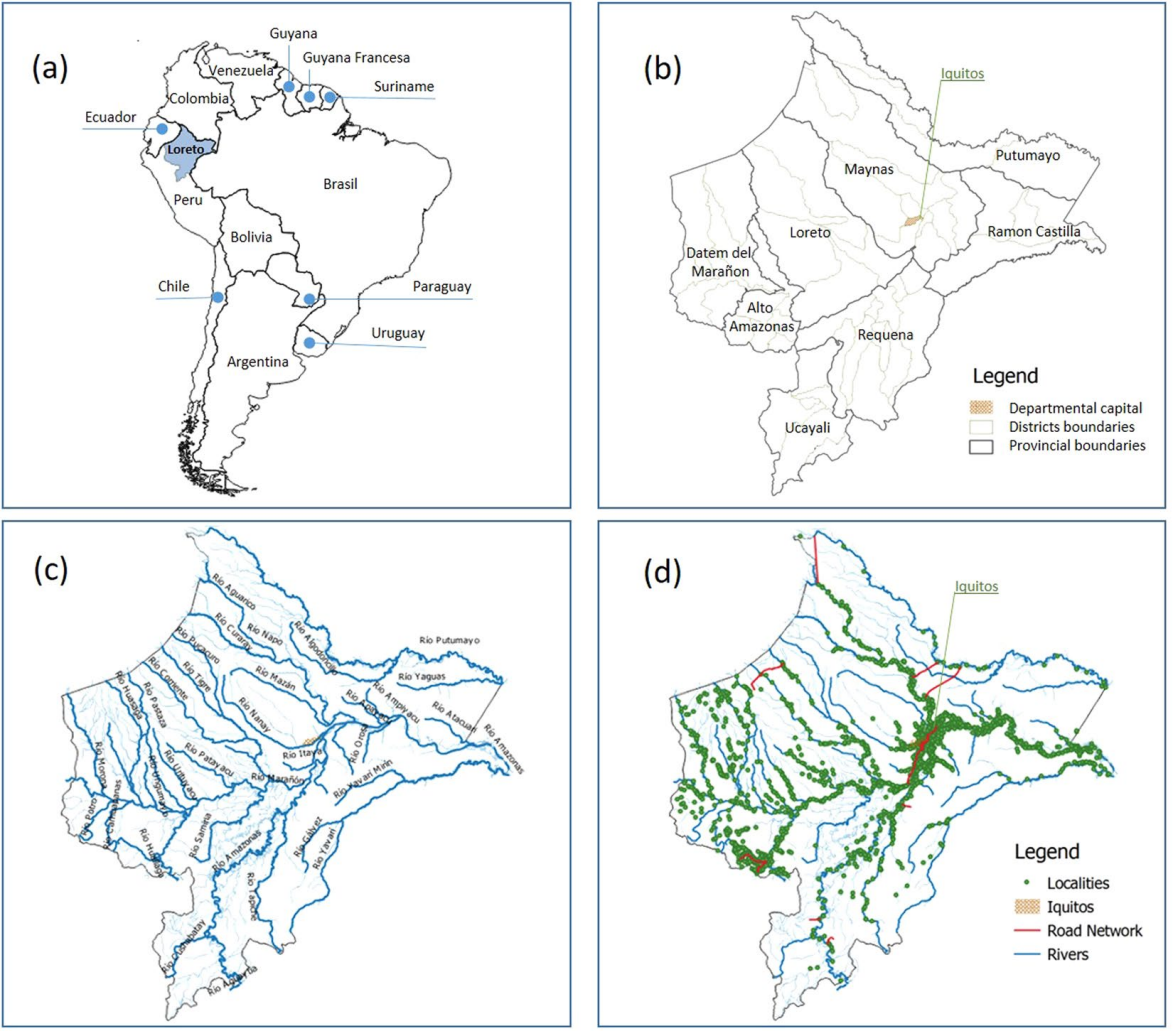
Study area: (**a**) geographical location of Loreto in South America; (**b**) administrative division of Loreto: department, provinces, and districts; (**c**) hydrographic map of Loreto; (**d**) road network, rivers, and georeferenced villages.

Loreto, located in the northeast part of Peru between 61 to 220 meters above sea level, is the largest department in Peru (368,851.95 km2, 28.7% of the national territory). A total of 883,510 inhabitants live in the department, with ~40% of them aged below 15 years, and ~one third residing in rural areas^37^. Loreto has a tropical climate, with a rainy season from November to May and a dry season from June to October. Precipitation is present throughout the year (cumulative average: 2,500 mm) with a peak in March (360 mm) and a minimum in July (50–100 mm). Rural villages mainly depend on natural resource exploitation, while commercial activities are more important in cities and large villages. Loreto has an extensive fluvial network connected to the Amazon River (Fig. 1), and boats are the primary mode of transportation.

### Population and malaria incidence

Of the 2,843 villages included in the official cartography of Loreto, we validated and georeferenced 2,766 villages in Loreto as points in QGIS^38,39^. Non-validation was primarily due to duplicated names and/or duplicated coordinates. The population of villages, obtained from the exceptional National Census of 2012–2013^40^, was assumed to be uniform during the study period.

Malaria is a mandatory notifiable disease in Peru. Malaria cases are confirmed by light microscopy in health facilities and attributed to the apparent place of infection using a village’s alphanumeric code, then reported to the surveillance system of Ministry of Health (MoH)^41^. Since malaria has been associated with travel- and forest-related activities in Loreto, the place of infection provides a more accurate information than the place of residence when modelling malaria transmission. For the purpose of our study, all reported cases in Loreto between 2010 and 2017 were aggregated by village and by year. Yearly overall and species-specific indices (API) were calculated for each village using the formula: (number of confirmed cases/village population size) x 1,000. Villages were classified as being or not at high-risk (i.e. API > 10 cases/1,000 people) or very-high-risk (API > 50 cases/1,000 people) based on MoH thresholds.

### Social and environmental predictors

Social and environmental variables previously associated with malaria transmission^7,42,43^ were tested as factors for high-risk (IPA > 10) or very-high-risk (IPA > 50) (Table 1). These variables were derived from different satellite imagery and mixed products. JavaScript codes for Google Earth Engine (GEE)^44^ were used to download and process this images at Datum WGS84 zone 18 (EPSG 4326). Below is a brief description of the variables:

**Table 1:** Predictor and outcome variables used in BRT models.

Variable description					Source information				
Type	Variable	Descriptions	Units	Time-dependent variable	Data collection	Source	Spatial resolution	Temporal resolution	Units
Environment predictor variable	FC	Forest cover in a 2-km side square grid around village	%	yes (year)	UMD/hansen/global_forest_change_2015	Univ. Maryland	30 m	year	% per output grid cell
	FL	Annual forest loss in a 2-km side square grid around village	%	yes (year)	UMD/hansen/global_forest_change_2015	Univ. Maryland	30 m	year	% per output grid cell
	CAR	Cumulative annual rainfall. (average in a 2-km side square grid around village)	mm	yes (year)	TRMM/3B42	TRMM	~27 km	3hrs	mm/3hrs x pp
	LST	Land surface annual mean temperature	°C	yes (year)	MODIS/006/MOD11A1/LST_Day_1 km	NASA, MODIS, LST	1km	1 day	°C
	NDVI	Normalized difference vegetation index. (average in a 2-km side square grid around village)	index	yes (year)	MODIS/006/MOD13Q1	NASA, MODIS, Vegetation Index	250 m	8 days	index
	NDWI	Normalized difference water index. (average in a 2-km side square gride around village)	index	yes (year)	LANDSAT/LC05C01/T1_T1 LANDSAT/LC08/C01/T1_T1 LANDSAT/LE07/C01/T1_T1	NASA, LANDSAT	30 m	16 days	index
	SDR	Euclidean shortest distance to rivers	kilometers	no	JRC/GSW1_0/GlobalSurfaceWater (occurrence)	JRC/GSW Historical data	30 m	once	kilometers
Social predictor variable	TPV	Travel time to major populated villages/towns	minutes	no	Oxford/MAP/accessibility_to_cities_2015_v1_0	Oxford (MAP), Google, (JRC) & Univ. Twente	1Km	once	minutes
	POPD	Population in a 5-km side square grid around village)	log (number people)	no	WorldPop/POP	WorldPop 2015	~100 m	once	people in ~100 × 100 m grid cell
Outcome variable	Malaria high-risk	Village with API** > 10 cases/1000 people	binary (yes/no)	yes (year)	—	Surveillance system of Peruvian ministry of health	—	week	reported cases
	Malaria very-high-risk	Village with API > 50 cases/1000 people	binary (yes/no)						

**API = Confirmed cases in a year *1000 / total population.

*PP: Per-pixel.

*Time to major populated villages/towns (TPV)*, social variable indicating the land-based travel time in minutes to the nearest densely-populated area for the year 2015. Underlying datasets included roads, railways, rivers, lakes, oceans, slope and elevation, land cover types, and national borders^22^.

*Population density (POPD)*, social variable representing the estimated number of residents in each 100mx100m grid-cell in 2015, extracted from WorldPop (https://www.worldpop.org)^29^.

*Shortest distance to rivers (SDR)*, environmental variable estimated using the Global Surface Water (occurrence) map^45^ from JRC (Joint Research Centre). A 50% threshold mask was applied in GEE to select pixels with a presence of water at least half of the period 1984–2015, capturing main and secondary rivers. The shortest distance in (meters) between rivers and villages was calculated using the proximity algorithm QGIS v.3.4.2^46,47^.

*Forest coverage (FC)* and *forest loss (FL)*, time-dependent measures of the area covered by trees (%) in each year and the loss of tree coverage compared to the previous year (%), calculated from 2000–2018 Global Forest Change data (https://earthenginepartners.appspot.com/science-2013-global-forest/download_v1.6.html)^48^.

*Cumulative annual rainfall (CAR)*, the estimated cumulative yearly rainfall (mm/year) calculated from daily 3-hour infrared precipitation estimates in product 3B42 of the Tropical Rainfall Measuring Mission (TRMM)^49^.

*Normalized Difference Water Index (NDWI)*, a satellite-derived index from the Near-Infrared (ρNIR) and Short Wave Infrared (ρSWIR) channels that estimates the amount of water in internal leaf structure^50,51^. This index was processed from Landsat 5, 7 and 8 T1 (calibrated TOA, DOS and clouds masking), and calculated as NDWI = (ρNIR-ρSWIR)/(ρNIR + ρSWIR)^52,53^.

*Normalized Difference Vegetation Index (NDVI)*), the estimated fraction of radiation absorbed by the vegetation in the red (ρRED) and the near infrared (ρNIR) channels^54^, from the MODIS/006/MOD13Q1 Moderate Resolution Imaging Spectroradiometer - National Aeronautics and Space Administration (NASA). The index was calculated at a spatial resolution of 250 m. as: NDVI = (ρNIR-ρRED)/(ρNIR + ρRED)^52,55–57^.

*Land Surface Temperature (LST)*, an estimate of the infrared emissivity of the earth in degrees Celsius (Emissivity Daily L3 Global, MOD11A1 version 6)^58^. Mean values from 365 daily layers were used to produce raster images with yearly means.

### Extraction of values

Raster images were re-sampled to 90-meter-pixels using R software version 3.5.1. The focal function generates an output raster based on the neighborhood information^59^ using “moving windows” around pinpointed villages. It was used here to capture a 2 km-side square grid for FC, FL, CAR, NDVI, NDWI and LST, and a 5 km-side square grid for POPD. The 2 km-side threshold was chosen to cover breeding and resting sites within the flight distance for *An. darlingi* mosquitoes^60,61^, while a 5 km-side threshold capturing the population within and around the village. Raster values in square grids were aggregated as averages for environmental variables and as sums for POPD. For TPV and SDR, the extracted values were at village location. Corresponding R codes are presented in Supplementary Text S1.

### Boosted regression tree analysis

BRT models^62,63^ were created using R packages “gbm”^64,65^ and “dismo”^31^ to examine the relationship between potential predictors and the species-specific malaria risk status in villages for each study year. The binary data (i.e. villages with or without high-risk of malaria; or, villages with or without very-high-risk of malaria) was fitted using a Bernouilli response distribution. The optimal learning rate, bag.fraction and tree complexity were determined by testing several combinations with the lowest model deviance. Ten-fold cross-validation procedures using the “gbm.step” function enabled the selection of the optimal number of regression trees for each model, ensuring the same villages in cross-validation groups across models with the parameter “fold.vector”.

The relative contribution (RC) percentage was used to assess the relevance of each variable in BRT models. This metric measures how often the predictor is selected for partitioning, weighted by the squared model improvement resulting from successive partitions^31,66^. Partial dependence plots were generated to describe the effect of one predictor on the malaria risk status of the villages, after accounting for the average effects of all other predictors^67^. In these plots, the vertical axis is the logit of cross-validated predicted probability (logit (p)) for high-risk or very-high-risk of malaria, and the horizontal axis is the variable predictor with corresponding units.

The area under the curve (AUC) of the receiver operating characteristic (ROC) curve assessed the performance of BRT models for discriminating the malaria risk status in villages. Each cross-validation BRT model built with data of a given year yielded a cross-validated AUC (cvAUC), while its model predictions (i.e. predicted probabilities for villages at malaria risk) with testing data of the following year allowed for the estimation of a testing AUC (tAUC).

### Risk maps elaboration

BRT models built with 2016 data were tested with 2017 data, obtaining predicted probabilities for villages at high-risk (API > 10) and villages at very-high-risk (API > 50). These predicted probabilities were then used to classify villages into four categories (i.e. 0–0.25, 0.251–0.50, 0.511–0.75, 0.751–1.00), and to create species-specific risk maps for 2017 using QGIS v.3.4.2-Madeira.

### Ethical considerations

This study is part of the research project “Spatial tools and optimization techniques to support case detection strategies for the malaria control in the Peruvian Amazon” approved by Universidad Peruana Cayetano Heredia, SIDISI number 101052.

## Results

### Malaria occurrence

Of the 321,210 malaria cases reported to the epidemiological surveillance system in Loreto between 2010 and 2017, 311,128 cases (96.9%) occurred among our validated and georeferenced 2,766 villages. Non-pinpointed cases (3.1%) could correspond to infections in transient populations and/or inaccurate records of the place of the infection.

**Figure 2:**
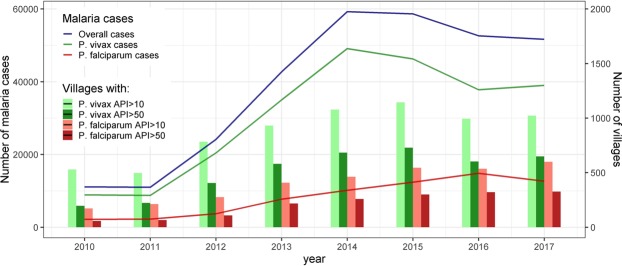
Reported malaria cases and number of villages at risk in Loreto from 2010 to 2017.

Malaria steadily increased in study villages from 10,994 cases in 2011 to 59,257 cases in 2014 and 58,679 in 2015, after which cases slightly declined to 51,663 in 2017 (Fig. 2). *P. vivax* cases always predominated over *P. falciparum* cases with the maximum ratio in 2012 (Pv/Pf 5.5) and the minimum ratio in 2016 (Pv/Pf 2.6). The highest peak occurred earlier for *P. vivax* (2014) than for *P. falciparum* (2016). The number of villages at high-risk (API > 10) and at very-high-risk (API > 50) followed similar trends.

### Relative contribution (RC) of variables

**Figure 3:**
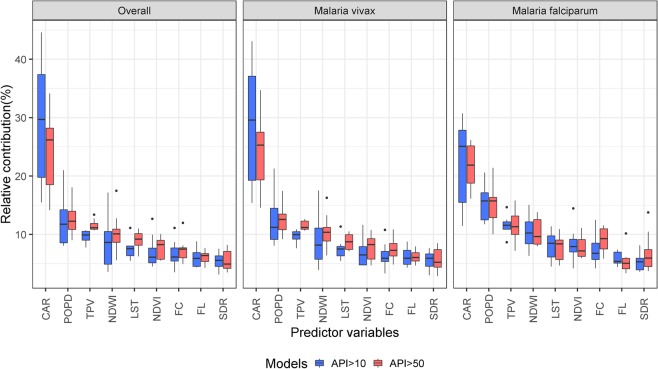
Relative contributions of predictors obtained from yearly BRT models for malaria risk, overall and by species, over the study period (2010–2017).

Mean (M), standard deviation (SD), median (Mdn), and interquartile range (IQR) values of predictor variables are presented in Supplementary Table S1. The RC of predictors obtained from yearly cross-validated BRT models are shown in Supplementary Tables S2, S3 and S4 and Fig. S1. Most RCs for overall malaria risk were similar to ones for *P. vivax* each year. CAR, POPD, and TPV, in that order, were always the top three predictors for increasing malaria risk in villages (overall and by species), with yearly RC medians exceeding 10% (Fig. 3). NDWI was the fourth top predictor, but yearly RC medians only exceeded 10% when BRT models assessed villages at *P. vivax* very-high-risk of and villages at *P. falciparum* high-risk.

CAR was generally the most relevant predictor in yearly BRT models for both *P. vivax* and *P. falciparum*, and presented the widest variation in RC. The analysis for high-risk in villages showed a higher RC of CAR for *P. vivax* than for *P. falciparum* (Supplementary Fig. S1), with the lowest RC for both species in 2010 and the highest in 2015 (RC range for *P. vivax*: 17%-48.4%; RC range for *P. falciparum*: 11.5%-30.7%). The analysis for very-high-risk found the lowest RC in 2010 for both *P. vivax* (14.3%) and *P. falciparum* (16.1%), and the highest in 2015 for *P. vivax* (35.5%) and in 2016 for *P. falciparum* (26.2%). The estimated RC for CAR followed similar trends with increasing importance of CAR as malaria risk predictor until 2015 for *P. vivax* and until 2015–2016 for *P. falciparum*, followed by a decrease (*P. vivax*) or stabilization (*P. falciparum*).

Unlike CAR, the POPD RC in villages was slightly higher for *P. falciparum* than for *P. vivax* in most years (Supplementary Fig. S1). The relevance of POPD varied widely across years only when BRT models assessed for *P. vivax* high-risk (range: 9.2–22.6%) and *P. falciparum* very-high-risk (range: 10.0–21.4%). The highest RCs for *P. vivax* malaria risk were found at the beginning of the study period (2010–2012) and then decreased and remained low with small variations; while RCs for *P. falciparum* increased from 2010 to 2014 and then declined in the following years.

The importance of NDWI as species-specific malaria risk predictor varied during the study period. The difference between lowest and highest RCs ranged between 3.8 and 18.9%, with an initial decrease in RC from 2010 to 2011 followed by peaks in 2012 and in 2016. The non-time dependent variable TPV was the predictor with the lowest RC variation across years for *P. vivax* risk in villages (7.4%-11.6% and 11%-13.1% for high-risk and very-high-risk respectively). It was also among the predictors with the lowest RC ranges when assessing the *P. falciparum* risk across time. For all other predictors in species-specific BRT models, the difference between lowest and highest RCs did not exceed 10% across years.

### Partial dependence plots

Supplementary Fig. S2 presents partial dependence plots (PDPs)^68^ of the marginal effect of predictor variables on the probability of villages to be at high-risk or very-high-risk, and shows whether the relationship is linear, monotonous or complex. The probability for a village to be at high-risk of *P. vivax* malaria generally increases with: CAR over 800 mm/3 hrs, POPD between 403 and 2,980 in the 5-km side square grid around the village, TPV superior to 700 minutes, NDWI around 0.4, FC higher than 50% in the 2-km side square grid around the village, NDVI between 0.7 and 0.8, LST higher than 27 °C, shorter SDR, and higher FL in the in the 2-km side square grid around the village. The probability for a village to be at high-risk for *P. falciparum* malaria increased with CAR over 800 mm/3 hrs, POPD between 403 and 1096 inhabitants, longer TPV, NDWI around 0.4, FC superior to 60%, NDVI ranging between 0.5 and 0.8, LST higher than 26 °C, shorter SDR, and higher FL. PDPs for very-high-risk are shown in Supplementary Fig S3.

### The discriminatory capacity of the models

The cvAUCs shown in Table 2 suggest that most yearly BRT models efficiently discriminate malaria risk in villages (cvAUC > 0.80), with the exception of the 2010 models for villages at high-risk (cvAUC = 0.72), and very-high-risk of *P. vivax* (cvAUC = 0.77), as well as *P. falciparum* high-risk (cvAUC = 0.78). The estimated tAUCs decreased slightly when the yearly models were tested with data corresponding to the following year, but most of the models still efficiently predicted malaria risk in the following year. For example, 2016 species-specific models using 2017 data were able to efficiently discriminate villages at high-risk (tAUC = 0.81) and very-high-risk (tAUC = 0.84) of *P. vivax*, and high-risk (tAUC = 0.83) and very-high-risk of *P. falciparum* (tAUC = 0.83) in 2017. Results from the discriminatory assessment of yearly BRT models with data from other years are presented in Supplementary Fig. S4.

**Table 2:** Assessment of the discriminating power of BRT models for malaria risk in villages.

	Model	Overall		*P. vivax*		*P. falciparum*	
		cvAUC	tAUC	cvAUC	tAUC	cvAUC	tAUC
High risk (API > 10)	2010	0.72	0.70	0.72	0.70	0.78	0.76
	2011	0.80	0.76	0.80	0.75	0.86	0.74
	2012	0.82	0.80	0.82	0.80	0.84	0.81
	2013	0.84	0.80	0.83	0.80	0.87	0.80
	2014	0.83	0.82	0.82	0.82	0.85	0.84
	2015	0.82	0.79	0.82	0.79	0.87	0.85
	2016	0.82	0.80	0.82	0.81	0.87	0.84
	2017	0.82	—	0.83	—	0.87	—
Very high risk (API > 50)	2010	0.76	0.76	0.77	0.76	0.82	0.78
	2011	0.85	0.81	0.84	0.80	0.88	0.81
	2012	0.85	0.82	0.85	0.81	0.87	0.76
	2013	0.86	0.80	0.86	0.80	0.89	0.84
	2014	0.84	0.84	0.84	0.83	0.89	0.83
	2015	0.85	0.83	0.85	0.82	0.89	0.86
	2016	0.84	0.83	0.84	0.83	0.88	0.83
	2017	0.85	—	0.84	—	0.89	—

Each cross-validation BRT model built with data of a given year yielded a cross-validated AUC (cvAUC), while its model predictions with testing data of the following year allowed for the estimation of a testing AUC (tAUC).

### Risk mapping

**Figure 4:**
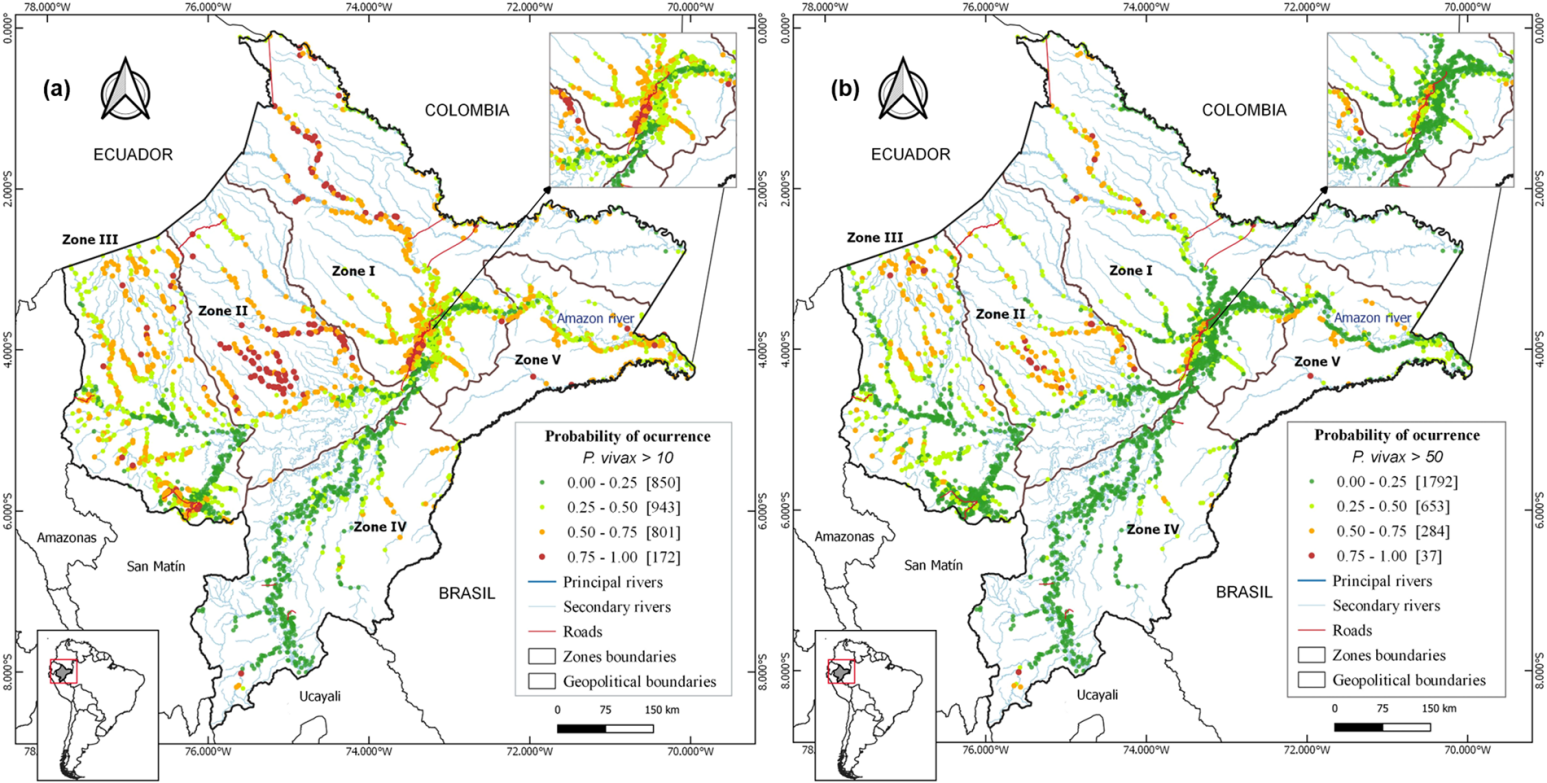
Predicted *P. vivax* risk maps for the year 2017 using 2016´s BRT models, showing: (**a**) villages at high *P. vivax* risk (API > 10 cases/1,000 people), (**b**) villages at very high *P. vivax* risk (API > 50 cases/1,000 people). Colors indicate the probability of a village (dots) of being at risk.

**Figure 5:**
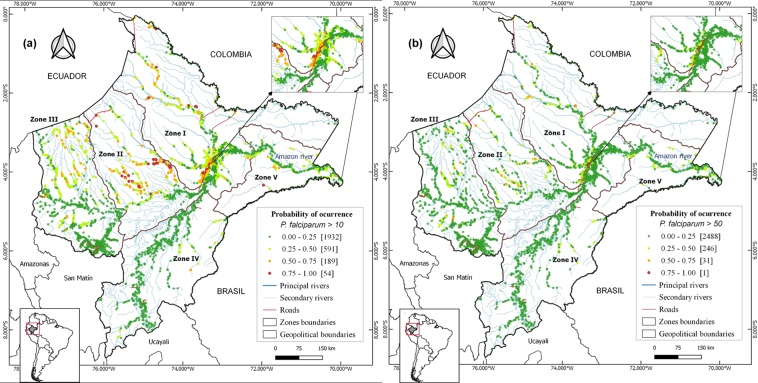
Predicted *P. falciparum* risk maps for the year 2017 using 2016´s BRT models, showing: (**a**) villages at high *P. falciparum* risk (API > 10 cases/1,000 people), (**b**) villages at very high *P. falciparum* (API > 50 cases/1,000 people). Colors indicate the probability of a village (dots) of being at risk.

The study villages and their malaria risk in 2017 (estimated from 2016 BRT species-specific models) were mapped (Figs 4 and 5) in five distinct zones based on contiguity between districts, main networks of transport, shared river basins, and population density.

*Zone I*, includes Maynas province and is the largest, most densely populated and accessible area in the department. The risk maps showed that 42.9% of villages were at high-risk of *P. vivax*, among which 10.5% were at very-high-risk. Also, 11.7% of villages were at high-risk of *P. falciparum*, among which 1.4% were at very-high-risk.

*Zone II*, covers Loreto province; it is the least populated area. Most villages (56.5%), were at high-risk of *P. vivax*, and 28.7% at very-high-risk. For *P. falciparum*, 27.8% were at high-risk, and 3.5% at very-high-risk.

*Zone III*, is the second most densely populated area and covers Datem del Maranon and Alto Amazonas provinces, which include 30% of the study villages and 19% of the province’s population. One third (34.5%) of the villages were at high-risk of *P. vivax*, and 12.3% at very-high-risk. For *P. falciparum*, 5.4% were at high-risk, and 1.1% at very-high-risk.

*Zone IV*, comprises Requena and Ucayali provinces, with 3.9% of villages at high-risk of *P vivax*, and 1.4% at very-high-risk. Only one village was at high-risk for *P. falciparum*.

*Zone V*, comprises Ramon Castilla province, and is the smallest zone, with 9% of the study villages and 6.7% of Loreto population. A significant proportion of villages (45.3%) were at high-risk of *P. vivax*, and 11.3% at very-high-risk. Seven villages were at high-risk of *P. falciparum*.

## Discussion

Using cross-validated BRT and remote-sensing (RS) satellite data, we modelled the distribution of malaria incidence by *Plasmodium* species in the Peruvian Amazon at village level between 2010 and 2017, and identified the most critical factors associated with this distribution. Yearly BRT models built with predictor data were able to efficiently discriminate the species-specific malaria risk in villages of the same year, and most of these models also performed well when predictor data was used to discriminate malaria risk in the following year. The high discriminatory capacity of the 2016 BRT models supported the generation of maps pinpointing villages with a high probability of having high malaria incidence (API > 10 cases x 1000 people) and very high incidence (API > 50 cases/1000 people) in 2017.

The mapping of malaria risk plays a key role in decision-making for designing and implementing malaria control measures^69^, but it requires the use of metrics that ensure an accurate description of malaria heterogeneity and the identification of changes in transmission intensity across time and geographical scales^70^. The entomological inoculation rate (EIR) and the parasite prevalence are the best metrics of malaria transmission intensity but their estimations require several community surveys per year (to capture annually seasonal variations), very large sample sizes, and specialized laboratory techniques, making them cost-prohibitive for National Malaria Control Programs (NMCPs)^70^. Instead, NMCPs have used the incidence of microscopically confirmed malaria cases detected by routine surveillance to map the risk of malaria transmission, prioritize intervention areas, and monitor the impact of control interventions^71^. Our study confirmed that predicted malaria risk from BRT models built with remote sensing derived predictors can accurately characterize the spatial distribution of the malaria incidence in Loreto.

The ability of *P. vivax* parasites to relapse from persistent liver parasite stages (hypnozoites) weeks or months after a primary parasitaemia^72^ may explain the lower discriminatory efficiency of *P. vivax* models compared with *P. falciparum* models in most study years. BRT models were built with available data from predictor variables reported to affect the malaria transmission. However, such variables are not yet proposed in the literature to be primary triggers of *P. vivax* relapses^72^. Once factors determining the hypnozoite reactivation are clearly identified, they could be incorporated to improve discriminatory capacity of the models for *P. vivax* risk. Improved models would be useful in the Peruvian Amazon, where epidemiological studies and parasite genotyping analyses suggest that relapses contribute substantially to the burden of *P. vivax* infections^2^. Moreover, data on main control interventions could also be used during the model building process, to account for the greater resilience of *P. vivax* to malaria control efforts in comparison with *P. falciparum*^73^.

Environmental factors such as temperature and rainfall directly affect the lifecycles of both parasite and vector; higher temperatures accelerate parasitic *plasmodium* growth within mosquitoes, while rainfall contributes to the accumulation of stagnant water that is ideal for mosquito breeding^61^. Results from partial dependence plots showing higher malaria risk when the yearly average LST ranged between 26 °C and 29 °C are in good agreement with the reported optimal temperatures for the development of the exogenous *P. vivax* and *P. falciparum* cycles within the main malaria vector *An. darlingi*^61^. However, due to the low variability of LST across villages, this variable was not among the most important factors for discriminating the malaria risk between villages. Instead, the variable satellite-derived rainfall was the best predictor for both species, with increased importance each year until2015. Unusually heavy rains in the last trimester of each year between 2011 and 2014 generated abrupt increases of river levels, and flooded to villages along the Amazonas River and its tributaries between 2012 and 2015^2,74–76^. The river levels not only peaked earlier and higher compared to historical averages, but also they remained high for a longer period. Levels of Amazon river in 2015 exceeded the threshold for imminent flooding (117 metres above sea level) from March through June^77^. River levels in 2011, 2012 and 2016 surpassed that threshold during 5, 14, and 4 weeks, respectively.

The proximity to breeding sites is an important determinant for the heterogeneity of both mosquito exposure and malaria occurrence in the Peruvian Amazon^78^. After seasonal rains, permanent breeding sites around villages become more productive, and additional breeding sites arise^79^. With severe flooding, breeding sites further enlarge and remain longer, leading to a wider dispersal of *An. darlingi* and increased vector-human contact rates. Villages with shorter distances to rivers had increased malaria receptivity and consequently, more malaria incidence as shown in PDPs. Increased vector contact would explain both the higher contribution of CAR in *P. vivax* and *P. falciparum* models and the better discriminatory efficiency of *P. vivax* models in the years of severe floods (2012–2015). Therefore, our findings suggest that new infections contributed more than hypnozoite-triggered relapses to the rise in the *P. vivax* malaria incidence since 2012.

In the literature, NDWI has been successfully used to identify bodies of water^80^. This predictor was an important factor in BRT models for both species (median in the study period slightly lower than 10%); but unlike with rainfall, its relative contribution did not present any identifiable temporal trend during the study period. Yearly average NDWI values around 0.4 were associated with increased malaria risk, suggesting that most of the 2-km side square grid around villages with high malaria incidence was covered by open water and/or wetlands, although NDWI did not allow to characterize those water bodies that are suitable for the development of *An. darlingi*. A recent study conducted in rural villages in the Peruvian Amazon found that the *An. darlingi* larval habitats were significantly associated with water bodies in landscapes with more recent deforestation, lower light intensity, emergent vegetation and a lower vegetation index^78^. The vegetation covering and surrounding these breeding sites could provide food for larvae, shelter from predators, and favourable oviposition conditions^81^. The yearly average NDVI values in the 2-km side square grid around villages would not differentiate this vegetation as suggested by the low contribution of this variable to the malaria risk models, and the increased malaria incidence associated with NDVI values above 0.6.

Deforestation and environmental changes driven by human activity have been associated with *An. darlingi* breeding and malaria transmission^16^; however, the heterogeneous definitions of deforestation in these studies precludes us from drawing a firm conclusion^17^. According to our BRT models, substantial recent tree cover loss in a 2-km side square grid around villages, forest loss was associated with higher malaria occurrence. However, this forest-related variable was not among the top predictors for discriminating the malaria risk, likely due to its limited variability across villages. The positive relationship between yearly deforestation and malaria risk is in line with entomological studies showing that *An. darlingi* larvae were more likely to be found in water bodies with recent deforestation^16,78^. Several studies in the Brazilian Amazon have found high densities of larval and adult *Anopheles* in forest fringes, as well as increased malaria morbidity in populations living or working near forest edges^82,83^. A relationship between forest coverage and forest edges might explain why villages with lower forest coverage have reduced malaria risk than those with higher coverage. As deforested areas increase, the distance to forest edges also increases but malaria transmission remains high because of the quantity and extension of forest edges around villages. Reduction in forest coverage would make forest edges more distant, hereby reducing malaria risk, unless residents engage in activities near forest edges. However, reduced forest coverage can also indicate increased socio-economic development, which is often associated with improvements in living conditions and malaria preventive practices^84^.

Vector-human contact requires that mosquitoes fly from breeding sites and forest edges to access to human blood meals^78^. Therefore, contact rates and malaria transmission strongly depend on both the dispersal of *An. darlingi* and the population density near breeding sites and forest edges. Our analysis highlighted the population density as the second most important predictor for malaria risk; yet, a positive relationship was only observed in the most densely populated villages. Further research will be required to confirm the negative relationship found in less densely populated villages. The sparsely distributed population in the Peruvian Amazon may be at increased malaria risk because of precarious conditions, with limited access to health care, and exposure to mosquito bites due to subsistence farming, fishing, hunting and other activities near or within the forest^18,19^. Besides, the time to major populated villages had a positive relationship with malaria risk, consistent with the idea that this variable can represent a valid proxy for diminished access to health care facilities, hereby reducing diagnosis and treatment, and hindering the delivery of malaria prevention interventions^21^.

The limitations of this study must also be acknowledged. First, the assumption of constant population size for villages across years could have reduced the discriminatory efficiency of the BRT models since they did not account for migration. Second, NDWI and NDVI might not be among the best proxies for environmental ground conditions that affect malaria transmission. Yearly means of these indexes might not capture the particular characteristics of breeding and resting sites of *An. darlingi*. Future research accounting for annual and seasonal variations in malaria risk and predictor variables will indicate to what extent predictive models can be improved. Third, population density within mosquito dispersal ranges does not only refer to the population living near breeding sites or forest edges, but also to individuals approaching these places for economic activities (human mobility). Unfortunately, the information was not available at the village level. Fourth, data of main control interventions per village during the study period was not available. These data would improve the discriminatory efficiency of models and allow a better assessment of the differential contribution of predictor factors between species. Fifth, the resampling of predictor raster data to a higher resolution always results in output raster as precise as the coarsest inputs, and it can add a systematic bias to developed models at high resolution. A higher resolution data for cumulative annual rainfall was not available for the study period. However, this variable has a low spatial variation in the Amazon region principally for its extensive plain topography^85^, and the corresponding bias is therefore expected to be limited.

In this study, we demonstrated that the probability of malaria occurrence in villages of Loreto in the Peruvian Amazon could be estimated using machine learning BRT models and RS-derived variables associated with the complex malaria host-vector-parasite relationships, resulting in predictive malaria risk maps that accurately characterized the spatial distribution of malaria incidence. Although further validation with better metrics of malaria transmission (i.e. EIR, parasite prevalence) is required, we hypothesize that the performance in discriminating the malaria transmission risk of model-predicted maps could surpass that of incidence maps in areas of reduced transmission and predominant asymptomatic infections not detected by traditional surveillance systems. This scenario will likely be reached shortly with the implementation of the 2017 governmental initiative “Zero Malaria Plan”^86^ aimed at reducing the burden of clinical malaria cases in the short-term, and eliminating the disease in the long-term.

## Supplementary information

SupplementaryMaterial_MalariaRiskMap

## Data Availability

The authors confirm that the data supporting the findings of this study are available within the article and/or its supplementary materials. Additional data is available from the corresponding author E.S-V. on request.
