# Supplementary material for: Malaria risk assessment and mapping using satellite imagery and boosted regression trees in the Peruvian Amazon
Source: Sci Rep. 2019 Oct 23;9:15173. doi: 10.1038/s41598-019-51564-4 (PMC6811674; doi:10.1038/s41598-019-51564-4)
Supplement: Supplementary file 1 — SupplementaryMaterial_MalariaRiskMap [file 41598_2019_51564_MOESM1_ESM.docx]

**Malaria risk assessment and mapping using satellite imagery and boosted regression trees in the Peruvian Amazon**

Elisa Solano-Villarreal*^1,2,3^, Walter Valdivia^4^, Morgan Pearcy^2^, Catherine Linard ^5,6^, José Pasapera-Gonzales^7^, Diamantina Moreno-Gutierrez^2,8,9^, Philippe Lejeune^1^, Alejandro Llanos-Cuentas^3^, Niko Speybroeck^2^, Marie-Pierre Hayette^1^, Angel Rosas-Aguirre^2,3,10^

1 University of Liege, 4000 Liege, Belgium.

2 Research Institute of Health and Society (IRSS), University catholique of Louvain, 1200 Brussels, Belgium

3 Institute of Tropical Medicine Alexander von Humboldt, Universidad Peruana Cayetano Heredia, Lima 15102, Peru

4 Ministry of Development and Social Inclusion, Lima 15047, Peru.

5 [Namur Research Institute for Life Sciences](https://www.narilis.be/) (Narilis), University of Namur, 5000 Namur, Belgium.

6 Institute of Life-Earth-Environment (ILEE), 5000 Namur, Belgium.

7 National Aerospace Development Commission, Lima 15046, Peru.

8 Centre for Health Economics Research and Modelling Infectious Diseases, Vaccine and Infectious

Disease Institute, University of Antwerp, 2000 Antwerp, Belgium.

9 Faculty of Human Medicine, National University of the Peruvian Amazon, Loreto 160, Peru.

10 Fund for Scientific Research FNRS, 1000 Brussels, Belgium

Elisa Solano-Villarreal

Email: elitayoan@gmail.com

Walter Valdivia

Email: Valdivia.w@gmail.com

Morgan Pearcy

Email: morgan.pearcy@uclouvain.be

Catherine Linard

Email: catherine.linard@unamur.be

Jose Pasapera-Gonzales

Email: josephjesus79@gmail.com

Diamantina Moreno-Gutierrez

Email: diamantina.moreno@uclouvain.be

Philippe Lejeune

Email: diamantina.moreno@uclouvain.be

Alejandro Llanos-Cuentas

Email: elmer.llanos@upch.pe

Niko Speybroeck

Email: niko.speybroeck@uclouvain.be

Marie-Pierre Hayette

Email: mphayette@chuliege.be

Angel Rosas-Aguirre

Email: angel.rosas@uclouvain.be

Fig. S1. Relative contributions (RCs) of predictors from yearly BRT models for malaria risk, overall and by species, over the study period (2010-2017): (a) high malaria risk (API>10 cases/1,000 people), (b) very high malaria risk (API>50 cases/1,000 people).

**(a)**


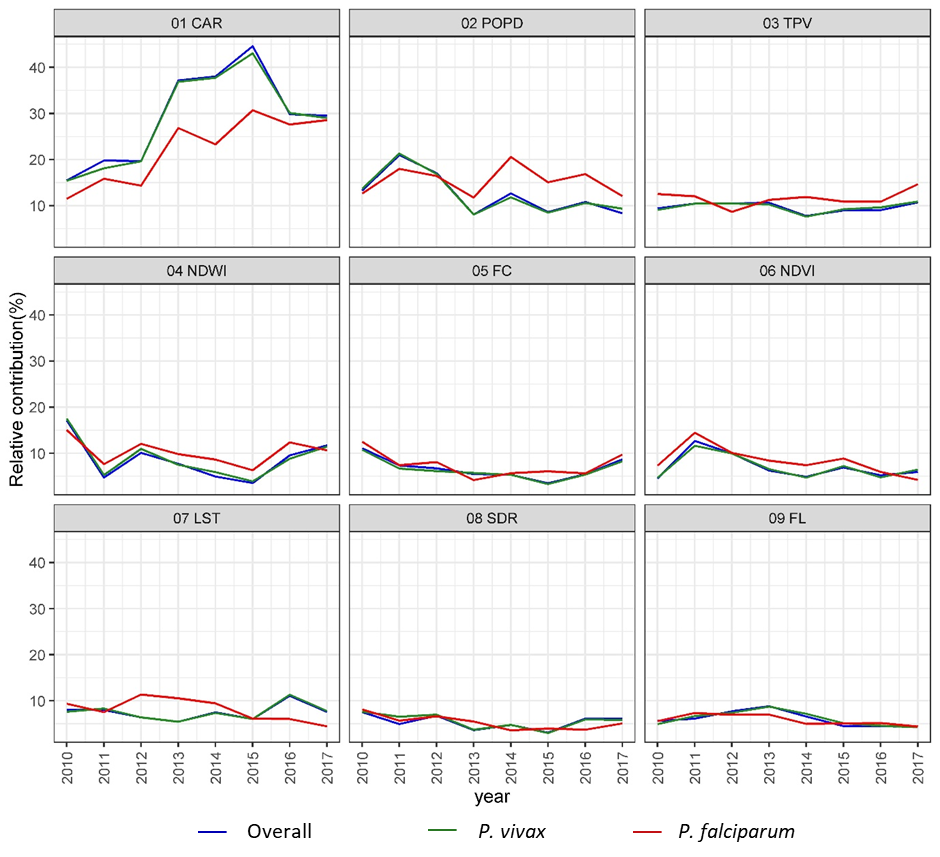


**(b)**


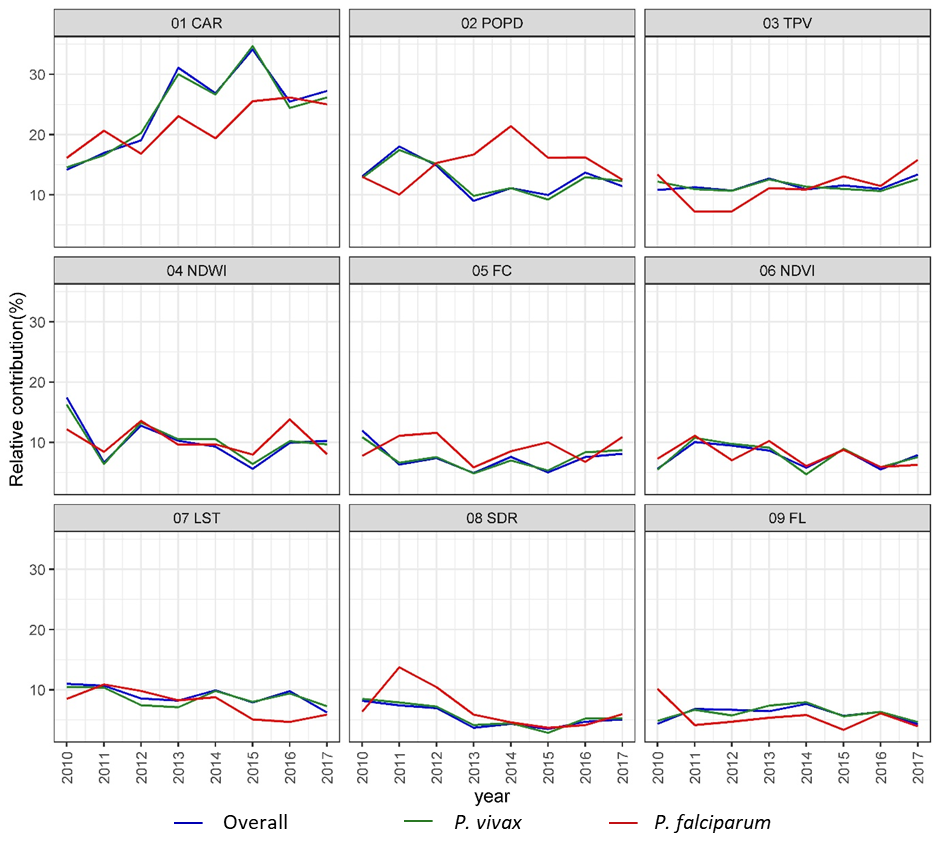


Figure S2. Partial dependence plots indicating the marginal effect of predictor variables on the probability of villages for being a high malaria risk (API>10 malaria cases/1,000 people): (a) *P. vivax* (b) *P. falciparum.*  Y-axes are on a logit scale. Red lines represent the predictions for 2017, while gray lines for other years. The distribution of variable values is indicated at the bottom of each plot.

**(a)**

**
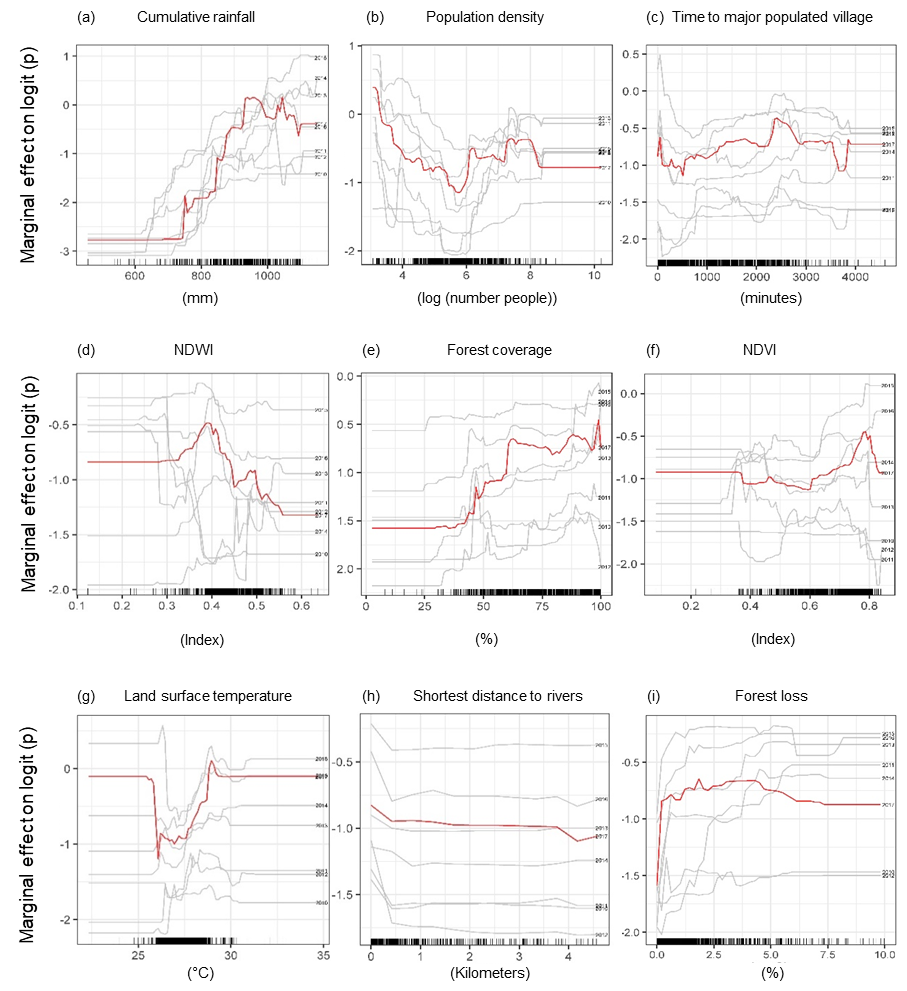
**

**(b)**

**
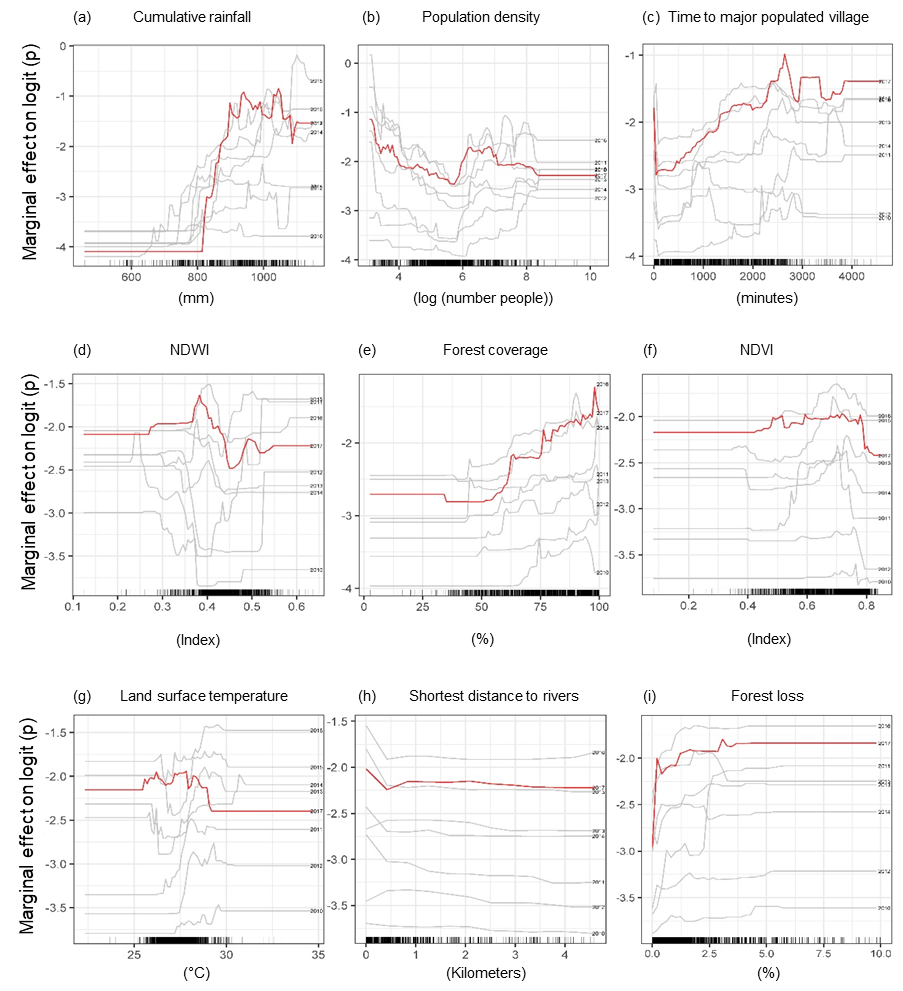
**

Figure S3. Partial dependence plots indicating the marginal effect of predictor variables on the probability of villages for being a very high malaria risk (API>50 malaria cases/1,000 people): (a) *P. vivax* (b) *P. falciparum.* Y-axes are on a logit scale. Red lines represent the predictions for 2017, while gray lines for other years. The distribution of variable values is indicated at the bottom of each plot.

**(a)**

**
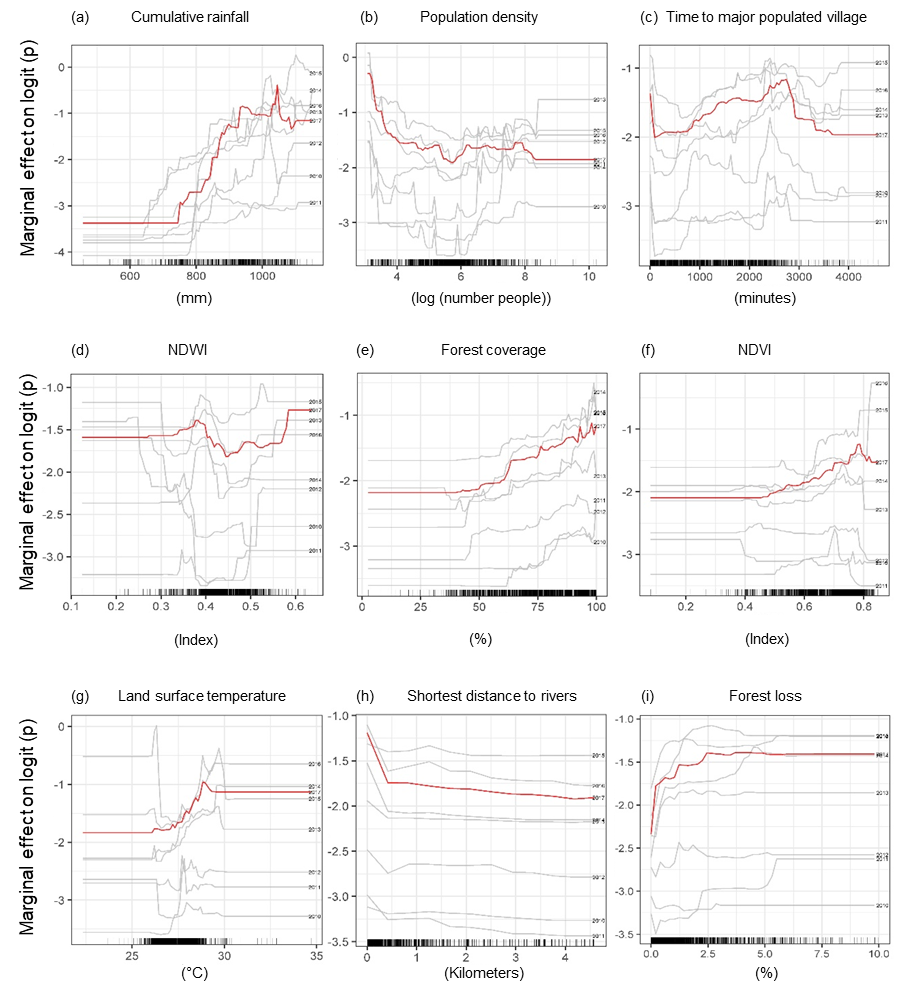
**

**(b)**

**
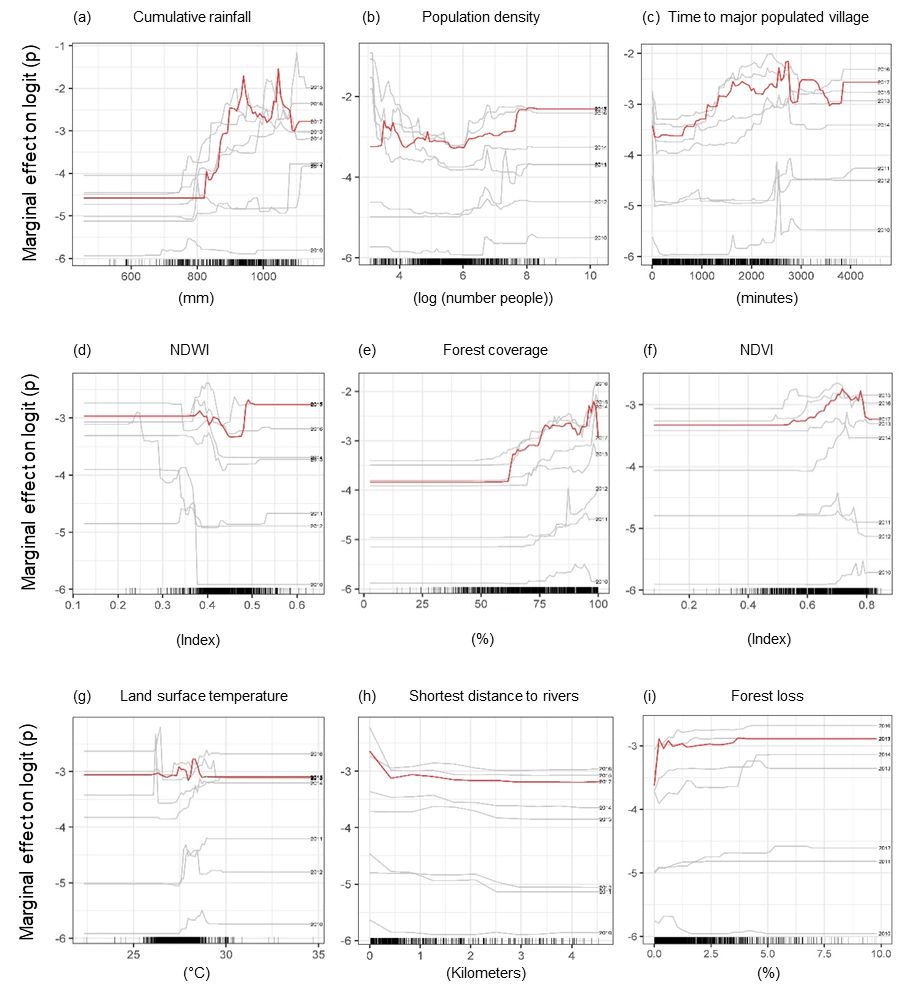
**

Figure S4: Discriminatory assessment of yearly BRT models with data from other years. The discriminating efficiency is assessed with the area under the ROC curve (AUC).

**
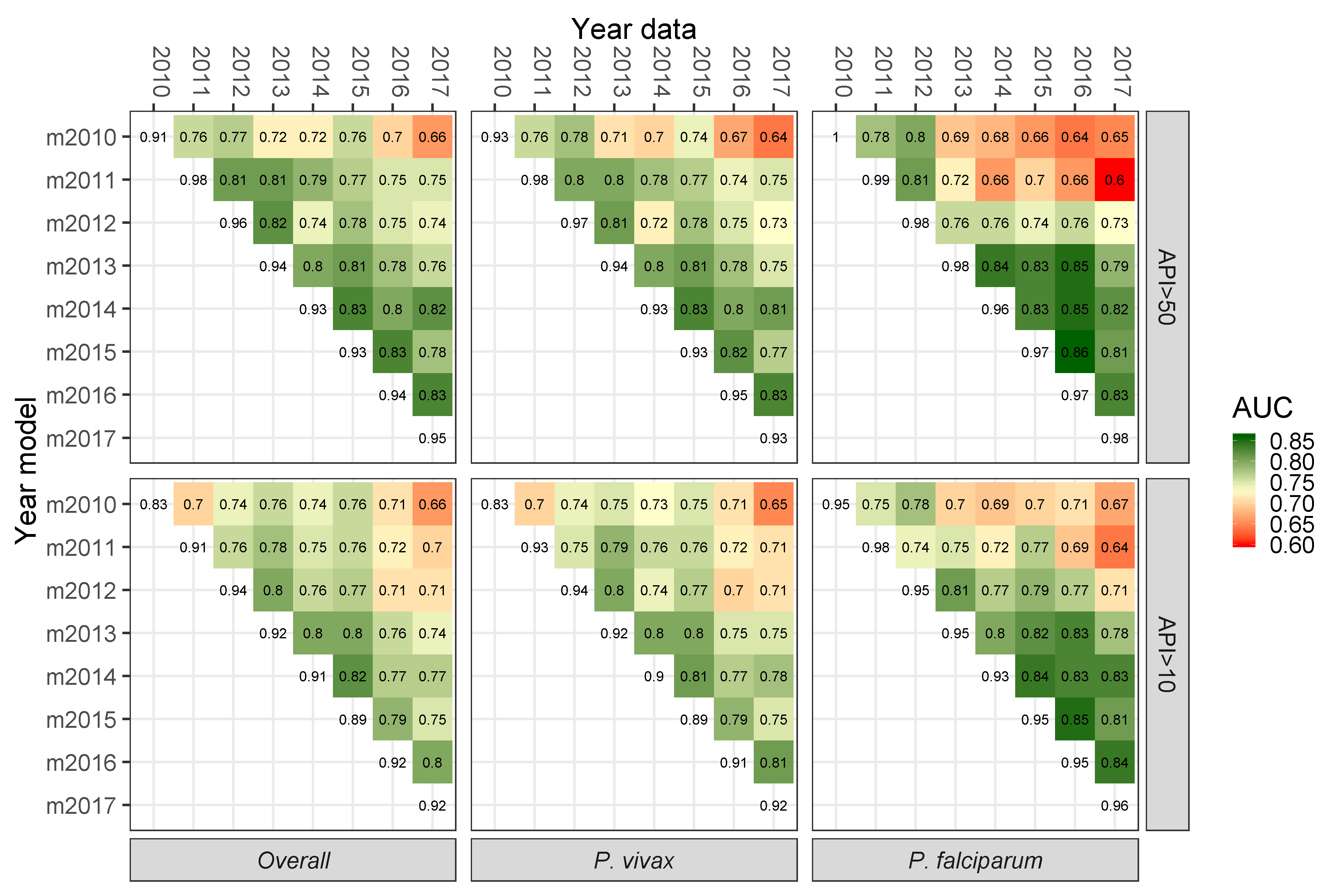
**

Table S1. Mean (M), standard deviation (SD), median (Mdn), and interquartile range (IQR) values of relative contributions of predictors for overall malaria, *P. vivax* and *P. falciparum* risk, over the study period (2010-2017).

| Predictors | Means   (M) | | Standard Deviations  (SD) | Medians   (Mdn) | Interquartile Ranges  (IQR) |
| --- | --- | --- | --- | --- | --- |
| CAR | 868.80 | 151.33 | | 876.80 | 212.21 |
| POPD | 475.27 | 997.32 | | 289.96 | 267.26 |
| TPV | 1236.28 | 902.57 | | 1111.25 | 1508.26 |
| NDWI | 0.41 | 0.05 | | 0.40 | 0.06 |
| FC | 77.05 | 16.26 | | 79.10 | 24.57 |
| NDVI | 0.67 | 0.11 | | 0.69 | 0.15 |
| LST | 27.76 | 0.93 | | 27.74 | 1.14 |
| SDR | 2.12 | 4.74 | | 0.30 | 1.28 |
| FL | 1.20 | 1.56 | | 0.67 | 1.35 |

Table S2. Relative contributions (RCs) of predictors from yearly BRT models for the overall malaria risk over the study period (2010-2017).

| **Risk** | **Variables** | **2010** | **2011** | **2012** | **2013** | **2014** | **2015** | **2016** | **2017** |
| --- | --- | --- | --- | --- | --- | --- | --- | --- | --- |
| **High risk (API>50)** | CAR | 16.3 | 20.8 | 20.4 | 39.1 | 41.3 | 49.3 | 31.8 | 32.6 |
|  | POPD | 14.7 | 22.8 | 17.7 | 8.8 | 13.5 | 9.8 | 12.8 | 9.2 |
|  | TPV | 10.4 | 10.6 | 11.1 | 12.1 | 8.0 | 9.7 | 9.2 | 10.6 |
|  | NDWI | 18.9 | 4.8 | 10.4 | 7.9 | 4.8 | 3.4 | 10.1 | 12.7 |
|  | FC | 12.3 | 7.4 | 6.8 | 5.8 | 5.5 | 3.3 | 6.0 | 8.7 |
|  | NDVI | 4.4 | 13.6 | 10.9 | 6.5 | 5.0 | 7.2 | 5.1 | 6.6 |
|  | LST | 8.8 | 8.4 | 6.8 | 5.6 | 7.7 | 6.5 | 11.5 | 7.6 |
|  | SDR | 8.3 | 5.0 | 7.2 | 4.1 | 5.5 | 3.7 | 7.0 | 6.3 |
|  | FL | 5.8 | 6.6 | 8.8 | 10.1 | 8.8 | 7.0 | 6.4 | 5.8 |
| **Very high risk (API>50)** | CAR | 14.4 | 17.1 | 20.4 | 32.0 | 29.0 | 36.5 | 26.4 | 27.2 |
|  | POPD | 13.8 | 19.5 | 15.3 | 9.5 | 11.9 | 11.0 | 15.1 | 12.6 |
|  | TPV | 10.7 | 12.7 | 11.5 | 13.3 | 11.2 | 12.8 | 11.5 | 13.4 |
|  | NDWI | 17.3 | 7.4 | 13.1 | 10.7 | 9.7 | 6.2 | 10.5 | 10.4 |
|  | FC | 12.5 | 6.8 | 6.8 | 5.1 | 7.9 | 5.4 | 7.8 | 8.4 |
|  | NDVI | 5.9 | 10.2 | 9.7 | 9.4 | 5.8 | 9.4 | 5.6 | 8.3 |
|  | LST | 10.9 | 11.0 | 9.0 | 8.4 | 10.5 | 8.2 | 10.5 | 7.0 |
|  | SDR | 9.3 | 8.1 | 7.1 | 4.6 | 5.1 | 3.5 | 5.1 | 6.2 |
|  | FL | 5.2 | 7.1 | 7.1 | 7.2 | 8.9 | 6.9 | 7.5 | 6.4 |

Table S3. Relative contributions (RCs) of predictors from yearly BRT models for the *P. vivax* malaria risk over the study period (2010-2017).

| **Risk** | **Variables** | **2010** | **2011** | **2012** | **2013** | **2014** | **2015** | **2016** | **2017** |
| --- | --- | --- | --- | --- | --- | --- | --- | --- | --- |
| **High risk (API>50)** | CAR | 17.0 | 18.5 | 20.6 | 38.1 | 41.8 | 48.4 | 33.2 | 31.7 |
|  | POPD | 14.9 | 22.6 | 17.8 | 9.2 | 13.0 | 9.4 | 12.2 | 10.4 |
|  | TPV | 10.3 | 11.3 | 11.0 | 11.6 | 7.4 | 9.7 | 10.3 | 11.1 |
|  | NDWI | 18.9 | 5.3 | 11.5 | 8.0 | 5.3 | 3.8 | 9.5 | 12.0 |
|  | FC | 11.9 | 7.1 | 6.3 | 5.8 | 5.4 | 3.1 | 5.5 | 8.2 |
|  | NDVI | 5.1 | 12.1 | 10.5 | 6.9 | 4.8 | 7.4 | 4.4 | 7.2 |
|  | LST | 8.2 | 9.3 | 6.5 | 5.7 | 7.3 | 6.8 | 11.7 | 7.6 |
|  | SDR | 9.0 | 6.5 | 7.3 | 4.3 | 5.4 | 3.5 | 6.6 | 6.2 |
|  | FL | 4.7 | 7.3 | 8.6 | 10.4 | 9.5 | 7.9 | 6.7 | 5.6 |
| **Very high risk (API>50)** | CAR | 14.3 | 17.3 | 19.9 | 30.8 | 27.7 | 35.5 | 25.6 | 28.1 |
|  | POPD | 12.9 | 18.7 | 15.5 | 10.2 | 12.1 | 10.3 | 14.2 | 14.1 |
|  | TPV | 12.0 | 12.0 | 11.0 | 12.7 | 11.5 | 12.5 | 11.0 | 13.1 |
|  | NDWI | 16.8 | 6.9 | 13.1 | 11.2 | 10.5 | 7.1 | 10.6 | 10.1 |
|  | FC | 12.2 | 7.1 | 7.8 | 5.6 | 7.7 | 5.4 | 8.4 | 8.2 |
|  | NDVI | 6.7 | 11.1 | 10.3 | 9.5 | 5.1 | 9.7 | 6.5 | 7.8 |
|  | LST | 11.6 | 11.1 | 8.3 | 7.3 | 10.5 | 8.8 | 10.2 | 6.8 |
|  | SDR | 9.1 | 8.7 | 7.8 | 4.6 | 5.1 | 3.7 | 6.0 | 5.9 |
|  | FL | 4.5 | 7.1 | 6.3 | 8.1 | 9.8 | 7.1 | 7.6 | 5.9 |

Table S4. Relative contributions (RCs) of predictors from yearly BRT models for the *P. falciparum* malaria risk over the study period (2010-2017).

| **Risk** | **Variables** | **2010** | **2011** | **2012** | **2013** | **2014** | **2015** | **2016** | **2017** |
| --- | --- | --- | --- | --- | --- | --- | --- | --- | --- |
| **High risk (API>50)** | CAR | 11.5 | 15.9 | 14.4 | 26.9 | 23.3 | 30.7 | 27.6 | 28.6 |
|  | POPD | 12.6 | 18.0 | 16.4 | 11.7 | 20.6 | 15.1 | 16.8 | 12.1 |
|  | TPV | 12.5 | 12.0 | 8.7 | 11.3 | 11.9 | 10.9 | 10.9 | 14.7 |
|  | NDWI | 15.1 | 7.6 | 12.1 | 9.8 | 8.7 | 6.3 | 12.4 | 10.6 |
|  | FC | 7.3 | 14.4 | 10.1 | 8.4 | 7.4 | 8.9 | 6.0 | 4.2 |
|  | NDVI | 9.4 | 7.6 | 11.4 | 10.5 | 9.5 | 6.1 | 6.1 | 4.5 |
|  | LST | 12.5 | 7.4 | 8.1 | 4.2 | 5.7 | 6.1 | 5.7 | 9.7 |
|  | SDR | 5.6 | 7.4 | 6.9 | 7.0 | 5.0 | 5.1 | 5.2 | 4.4 |
|  | FL | 8.2 | 5.7 | 6.6 | 5.5 | 3.6 | 4.0 | 3.7 | 5.1 |
| **Very high risk (API>50)** | CAR | 16.1 | 20.7 | 16.8 | 23.1 | 19.4 | 25.6 | 26.2 | 25.0 |
|  | POPD | 13.0 | 10.0 | 15.3 | 16.7 | 21.4 | 16.2 | 16.2 | 12.5 |
|  | TPV | 13.4 | 7.2 | 7.3 | 11.1 | 10.9 | 13.1 | 11.5 | 15.8 |
|  | NDWI | 12.2 | 8.4 | 13.6 | 9.6 | 9.7 | 8.0 | 13.8 | 8.1 |
|  | FC | 7.8 | 11.1 | 11.6 | 5.9 | 8.5 | 10.0 | 6.8 | 10.9 |
|  | NDVI | 7.3 | 11.1 | 7.0 | 10.2 | 6.0 | 8.7 | 5.9 | 6.3 |
|  | LST | 8.5 | 10.9 | 9.8 | 8.3 | 8.8 | 5.1 | 4.7 | 5.9 |
|  | SDR | 6.4 | 13.8 | 10.5 | 5.9 | 4.6 | 3.7 | 4.2 | 6.0 |
|  | FL | 10.2 | 4.2 | 4.7 | 5.4 | 5.8 | 3.4 | 6.1 | 3.9 |

Table S5. Number of villages at high malaria risk and at very high malaria risk according to predictions of 2016 BRT models using 2017 data. Malaria risk with predicted probabilities > 0.5.

|  |  | | **High risk (IPA>10)** | | | |  | | **Very high risk (IPA>50)** | | |
| --- | --- | --- | --- | --- | --- | --- | --- | --- | --- | --- | --- |
|  |  | | **n** | **N** | **%** |  | | **n** | | **N** | **%** |
| ***P. vivax*** | | Zone 1 | 369 | 860 | 42.9 |  | | 90 | | 860 | 10.5 |
|  | | Zone 2 | 179 | 317 | 56.5 |  | | 91 | | 317 | 28.7 |
|  | | Zone 3 | 294 | 853 | 34.5 |  | | 105 | | 853 | 12.3 |
|  | | Zone 4 | 19 | 489 | 3.9 |  | | 7 | | 489 | 1.4 |
|  | | Zone 5 | 112 | 247 | 45.3 |  | | 28 | | 247 | 11.3 |
| ***P. falciparum*** | | Zone 1 | 101 | 860 | 11.7 |  | | 12 | | 860 | 1.4 |
|  | | Zone 2 | 88 | 317 | 27.8 |  | | 11 | | 317 | 3.5 |
|  | | Zone 3 | 46 | 853 | 5.4 |  | | 9 | | 853 | 1.1 |
|  | | Zone 4 | 1 | 489 | 0.2 |  | | 0 | | 489 | 0 |
|  | | Zone 5 | 7 | 247 | 2.8 |  | | 0 | | 247 | 0 |

Text S1. The R script designed to resample raster image to 90-meter-pixels, and to compute average NDWI values in a 2km-side square grid, year 2010.

rm(list=ls())

gc()

library(raster)

library(rgdal)

library(data.table)

setwd("/home/users/a/r/arosas/ELISA/ndwi90/")

#setting an extent for Loreto raster (resolution 90 m)

ext<-raster("extension_NA.tif")

bb<-as.vector(extent(ext))

#call ndwi raster of year 2010 (resolution 30 m)

#cut the ndwi raster to obtain the same extension of Loreto raster

r2010<-raster("2010.tif")

r2010<-crop(r2010,bb)

#resampling of NDWI raster to 90 meters

rr2010 <- resample(r2010, ext,"bilinear")

###setting the 2 km-side in pixels (each pixel measures 90m)

b2k<-22

#focal function to capture information (mean values) in a 2 km-side square grid for NDWI

b10<- focal(rr2010, w=matrix(1,b2k,b2k), fun = mean, na.rm = TRUE)

###Generating the new raster

writeRaster(b10, "Ndwi10r90_b2x2.tif",overwrite=TRUE)
